# Dichlorido(3,5-dimethyl-1*H*-pyrazole)[(3,5-dimethyl-1*H*-pyrazol-1-yl)(*o*-tol­yl)methanone]palladium(II)

**DOI:** 10.1107/S1600536807066627

**Published:** 2007-12-18

**Authors:** Simphiwe M. Nelana, Gert J. Kruger, James Darkwa

**Affiliations:** a Department of Chemistry, University of Johannesburg, PO Box 524, Auckland Park, Johannesburg 2006, South Africa

## Abstract

In the title compound, [PdCl_2_(C_5_H_8_N_2_)(C_12_H_12_N_2_O)], the Pd atom adopts a slightly distorted *trans*-PdCl_2_N_2_ square-planar arrangement. The different Pd—N bond lengths can be related to the the electron-withdrawing effect of the *o*-toluoyl group on the substituted pyrazole ligand. The complex crystallizes as centrosymmetric hydrogen-bonded dimers through N—H⋯Cl linkages.

## Related literature

For related literature, see: Mukherjee (2000 ▶); Komeda *et al.* (2000 ▶); Li *et al.* (2002 ▶); Guzei *et al.* (2003 ▶); Guzei *et al.* (2005 ▶); Ojwach *et al.* (2005 ▶); Spencer *et al.* (2006 ▶); Allen (2002 ▶).
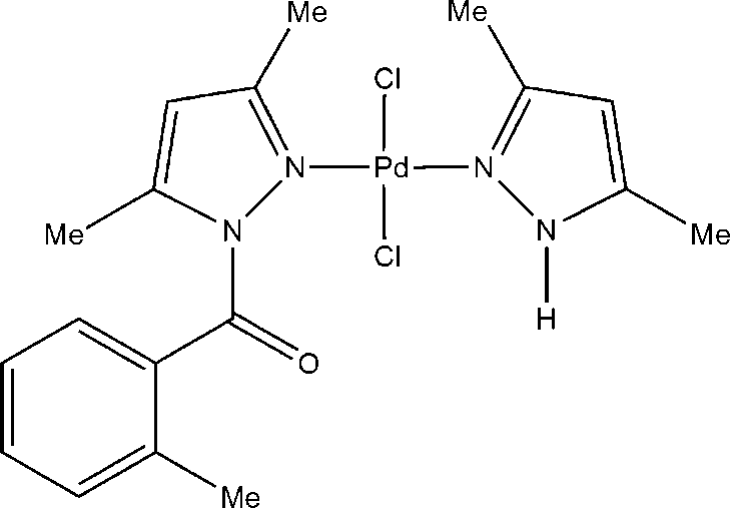


## Experimental

### 

#### Crystal data

[PdCl_2_(C_5_H_8_N_2_)(C_12_H_12_N_2_O)]
*M* *_r_* = 487.70
Orthorhombic, 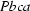

*a* = 15.908 (3) Å
*b* = 15.479 (3) Å
*c* = 16.602 (3) Å
*V* = 4088.0 (12) Å^3^
*Z* = 8
Mo *K*α radiation
μ = 1.18 mm^−1^
*T* = 293 (2) K
0.32 × 0.28 × 0.15 mm

#### Data collection

Bruker SMART CCD diffractometer
Absorption correction: multi-scan (*SADABS*; Sheldrick, 2004 ▶) *T*_min_ = 0.703, *T*_max_ = 0.843
43509 measured reflections
4023 independent reflections
2686 reflections with *I* > 2σ(*I*)
*R*_int_ = 0.056

#### Refinement

*R*[*F* ^2^ > 2σ(*F* ^2^)] = 0.040
*wR*(*F* ^2^) = 0.114
*S* = 1.08
4023 reflections
240 parameters
H-atom parameters constrained
Δρ_max_ = 1.09 e Å^−3^
Δρ_min_ = −0.49 e Å^−3^

### 

Data collection: *SMART-NT* (Bruker, 1998 ▶); cell refinement: *SAINT-Plus* (Bruker, 1999 ▶); data reduction: *SAINT-Plus*; program(s) used to solve structure: *SHELXS97* (Sheldrick, 1997 ▶); program(s) used to refine structure: *SHELXL97* (Sheldrick, 1997 ▶); molecular graphics: *ORTEP-3 for Windows* (Farrugia, 1997 ▶) and *Mercury* (Macrae *et al.*, 2006 ▶); software used to prepare material for publication: *WinGX* (Farrugia, 1999 ▶), *PLATON* (Spek, 2003 ▶) and *publCIF* (Westrip, 2008 ▶).

## Supplementary Material

Crystal structure: contains datablocks I, global. DOI: 10.1107/S1600536807066627/hb2672sup1.cif

Structure factors: contains datablocks I. DOI: 10.1107/S1600536807066627/hb2672Isup2.hkl

Additional supplementary materials:  crystallographic information; 3D view; checkCIF report

## Figures and Tables

**Table:** 

Pd1—N11	1.989 (4)
Pd1—N21	2.042 (4)
Pd1—Cl1	2.2981 (15)
Pd1—Cl2	2.3001 (15)

**Table:** 

N11—Pd1—N21	174.88 (15)
N11—Pd1—Cl1	88.39 (13)
N21—Pd1—Cl1	90.02 (12)
N11—Pd1—Cl2	89.98 (13)
N21—Pd1—Cl2	91.84 (12)
Cl1—Pd1—Cl2	176.71 (5)

**Table 2:** Hydrogen-bond geometry (Å, °)

*D*—H⋯*A*	*D*—H	H⋯*A*	*D*⋯*A*	*D*—H⋯*A*
N12—H12*A*⋯Cl2^i^	0.86	2.35	3.194 (4)	169

Symmetry code: (i) 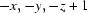.

**Fig. 1.**
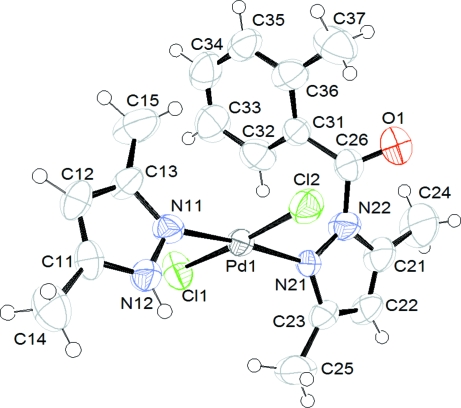
: The molecular structure of (I), with displacement ellipsoids drawn at the 50% probability level (arbitrary spheres for the H atoms).

**Fig. 2.**
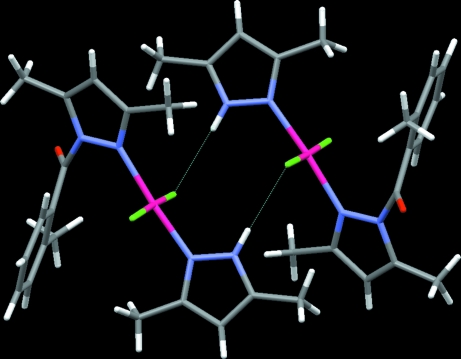
: A hydrogen-bonded dimer in (I) with the H bonds indicated by thin blue lines.

## supplementary crystallographic information

### Comment

Pyrazole and pyrazolyl ligands have been used to form N-donor metal complexes with interesting catalytic applications (Mukherjee, 2000) and as mimics for imidazole coordination in metalloenzymes (Komeda *et al.*, 2000). Catalytic behaviour of such N-donor metal complexes, in particular, depends on the nature of substituents on the pyrazolyl ligands. The introduction of dicarbonylbenzene linkers (Guzei *et al.*, 2003) to bis(pyrazole)palladium complexes (Li *et al.*, 2002), for instance, improves the activity of these complexes as ethylene polymerization catalysts. The presence of carbonyl functional groups in palladium complexes, however, reduces their stability as catalysts. We initially attributed the reduced stability to the effect of two carbonyl groups on the N-donor ability of the pyrazolyl ligands. In an attempt to improve the stability of the palladium catalysts, monocarbonylbenzene units were attached to pyrazolyl units to prepare the bis(pyrazolylcarbonylbenzene)palladium dichloride. Surprisingly, with (3,5-dimethyl-pyrazol-1-yl)-*o*-toluoyl-methanone as a ligand during complexation with PdCl_2_, we isolated the title compound, (I), a mixed ligand palladium complex, containing (3,5-dimethyl-pyrazol-1-yl)-*o*-toluoyl-methanone and 3,5-dimethylpyrazole as ligands. The formation of compound I appears to occur *via* partial hydrolysis of one of the (3,5-dimethyl-pyrazol-1-yl)-*o*-toluoyl-methanone ligands, presumably by traces of water in the reaction mixture.

Compound (I) displays square planar geometry around the palladium atom, with the two different pyrazolyl ligands ((3,5-dimethyl-pyrazol-1-yl)-*o*-toluoyl-methanone and 3,5-dimethylpyrazole) bonding *trans* to the metal centre *via* N atoms (Fig. 1). The slight distortion of the square planar configuration around the palladium atom can be seen in the differences in bond angles involving palladium (Table 1) and the deviations from the least-squares plane through the central five atoms [Pd1 = -0.0116 (12) Å, Cl1 = -0.0684 (12) Å, Cl2 = -0.0665 (12) Å, N11 = 0.0754 (17) Å, N21 = 0.0711 (16) Å], with the biggest deviations being observed for the nitrogen atoms. The pyrazolyl rings of the coordinating ligands are roughly perpendicular to this central plane, with interplanar angles of 86.66 (12)° and 68.13 (14)° respectively. The Pd—Cl bond distances in (I) are similar to Pd—Cl distances in related complexes (*e.g.* Spencer *et al.*, 2006) and consistent with the average of 2.33 (4)Å calculated for 2151 Pd—Cl distances in 1306 complexes reported to the Cambridge Structural Database (CSD, Version 5.26, updated May 2005; Allen 2002). One interesting feature of (I) is the significant difference of 0.053 Å in the Pd—N bond distances for the two ligands, illustrating the electron-withdrawing effect of the *o*-toluoyl-methanone substituent on the pyrazolyl ligand.

Intermolecular hydrogen bonding is responsible for the formation of centrosymmetric dimers through N—H···Cl linkages [Figure 2, Table 2]. In contrast to previous observations (Li *et al.*, 2002), the hydrogen bonding in (I) has no effect on the bond order: we observed two virtually identical Pd—Cl bond lengths, although only Cl1 was involved in a N—H···Cl hydrogen bond.

### Experimental

To a solution of [PdCl_2_(NCMe)_2_] (0.10 g, 0.47 mmol) in dichloromethane (30 ml), was added (3,5-dimethyl-pyrazol-1-yl)-*o*-toluoyl-methanone (0.06 g, 0.23 mmol). The formation of an orange-yellow solution was observed and the reaction mixture was stirred at room temperature for 24 h. The solution was then concentrated to 15 ml and an equal amount of hexane was added and kept at -4 °C for 3 days to yield yellow crystals suitable for X-ray analysis. Yield = 0.08 g, 59%. IR (Nujol): ν (C=O) = 1699. ^1^H NMR (CDC_l3_): δ 9.50 (s, 1H, N—H), 7.54 (m, 4H, *o*-toluoyl), 6.13 (s, 1H, 4-pz, *o*-toluoyl), 5.74 (s, 1H, 4-pz), 2.94 (s, 3H, *o*-toluoyl), 2.56 (s, 3H, 5-CH_3_, *o*-toluoyl), 2.35 (s, 3H, 3-CH*3*, *o*-toluoyl), 2.31 (s, 3H, 5-CH_3_,), 2.21 (s, 3H, 3-CH_3_). ^13^C{^1^H} NMR: δ 169.4, 157.4, 137.6, 133.3, 131.5, 128.4, 118.6, 106.5, 33.1, 32.4, 30.1.

### Refinement

The H atoms were geometrically positioned and refined in the riding-model approximation, with C—H = 0.93–0.96 Å, N—H = 0.86 Å, and *U*_iso_(H) = 1.2*U*_eq_(C) or 1.5*U*_eq_(N). The highest peak in the final difference map is 0.87 Å from Cl1 and the deepest hole is 0.88 Å from N11.

### Crystal data

**Table:**

[PdCl_2_(C_5_H_8_N_2_)(C_12_H_12_N_2_O)]	*F*_000_ = 1968
*M**_r_* = 487.70	*D*_x_ = 1.585 Mg m^−^^3^
Orthorhombic, *P**b**c**a*	Mo *K*α radiation λ = 0.71073 Å
Hall symbol: -P 2ac 2ab	Cell parameters from 2686 reflections
*a* = 15.908 (3) Å	θ = 2.2–26.0º
*b* = 15.479 (3) Å	µ = 1.18 mm^−^^1^
*c* = 16.602 (3) Å	*T* = 293 (2) K
*V* = 4088.0 (12) Å^3^	Block, yellow
*Z* = 8	0.32 × 0.28 × 0.15 mm

### Data collection

**Table:**

Bruker SMART CCD diffractometer	4023 independent reflections
Radiation source: fine-focus sealed tube	2686 reflections with *I* > 2σ(*I*)
Monochromator: graphite	*R*_int_ = 0.056
*T* = 293(2) K	θ_max_ = 26.0º
φ and ω scans	θ_min_ = 2.2º
Absorption correction: multi-scan(SADABS; Sheldrick, 2004)	*h* = −19→19
*T*_min_ = 0.703, *T*_max_ = 0.843	*k* = −18→19
43509 measured reflections	*l* = −20→20

### Refinement

**Table:**

Refinement on *F*^2^	Secondary atom site location: difference Fourier map
Least-squares matrix: full	Hydrogen site location: inferred from neighbouring sites
*R*[*F*^2^ > 2σ(*F*^2^)] = 0.040	H-atom parameters constrained
*wR*(*F*^2^) = 0.114	*w* = 1/[σ^2^(*F*_o_^2^) + (0.0425*P*)^2^ + 9.9045*P*] where *P* = (*F*_o_^2^ + 2*F*_c_^2^)/3
*S* = 1.08	(Δ/σ)_max_ = 0.001
4023 reflections	Δρ_max_ = 1.09 e Å^−^^3^
240 parameters	Δρ_min_ = −0.49 e Å^−^^3^
Primary atom site location: structure-invariant direct methods	Extinction correction: none

### Special details

**Table:**

Geometry. All e.s.d.'s (except the e.s.d. in the dihedral angle between two l.s. planes) are estimated using the full covariance matrix. The cell e.s.d.'s are taken into account individually in the estimation of e.s.d.'s in distances, angles and torsion angles; correlations between e.s.d.'s in cell parameters are only used when they are defined by crystal symmetry. An approximate (isotropic) treatment of cell e.s.d.'s is used for estimating e.s.d.'s involving l.s. planes.Atoms 'Deviations (Å)' Pd1 - 0.0116 (12) Cl1 - 0.0684 (12) Cl2 - 0.0665 (12) N11 0.0754 (17) N21 0.0711 (16)Atoms 'Deviations (Å)' N11 - 0.0012 (32) N12 0.0016 (33) C11 - 0.0010 (51) C12 - 0.0021 (49) C13 - 0.0038 (56) C14 0.0023 (42) C15 0.0042 (45)Atoms 'Deviations (Å)' N21 0.0327 (35) N22 - 0.0225 (38) C21 - 0.0159 (65) C22 0.0319 (54) C23 0.0057 (55) C24 - 0.0040 (49) C25 - 0.0279 (46)Atoms 'Deviations (Å)' C32 0.0353 (49) C31 0.0478 (44) C33 - 0.0055 (54) C34 - 0.0356 (52) C35 - 0.0064 (49) C36 0.0160 (51) C37 0.0075 (42) C26 - 0.0592 (38)'Plane 1' 'Plane 2' 'Interplanar Angle (°)' Square 'Ring 1' 86.66 (12) Square 'Ring 2' 68.13 (14) 'Ring 2' 'Ring 3' 55.98 (25)
Refinement. Refinement of *F*^2^ against ALL reflections. The weighted *R*-factor *wR* and goodness of fit *S* are based on *F*^2^, conventional *R*-factors *R* are based on *F*, with *F* set to zero for negative *F*^2^. The threshold expression of *F*^2^ > σ(*F*^2^) is used only for calculating *R*-factors(gt) *etc*. and is not relevant to the choice of reflections for refinement. *R*-factors based on *F*^2^ are statistically about twice as large as those based on *F*, and *R*- factors based on ALL data will be even larger.

### Fractional atomic coordinates and isotropic or equivalent isotropic displacement parameters (Å^2^)

**Table:**

	*x*	*y*	*z*	*U*_iso_*/*U*_eq_	
C11	0.1260 (3)	−0.2107 (3)	0.4805 (3)	0.0544 (13)	
C12	0.1495 (4)	−0.2290 (3)	0.4037 (4)	0.0588 (14)	
H12	0.1646	−0.2829	0.3836	0.071*	
C13	0.1468 (4)	−0.1521 (3)	0.3608 (3)	0.0551 (13)	
C14	0.1168 (4)	−0.2659 (4)	0.5543 (4)	0.087 (2)	
H14A	0.1677	−0.2977	0.5631	0.130*	
H14B	0.0709	−0.3054	0.5470	0.130*	
H14C	0.1057	−0.2297	0.6001	0.130*	
C15	0.1668 (5)	−0.1341 (4)	0.2743 (4)	0.095 (2)	
H15A	0.1195	−0.1067	0.2491	0.142*	
H15B	0.1791	−0.1874	0.2472	0.142*	
H15C	0.2148	−0.0966	0.2712	0.142*	
C21	0.1020 (4)	0.3084 (3)	0.3533 (4)	0.0726 (18)	
C22	0.1203 (4)	0.3102 (4)	0.4313 (4)	0.0668 (16)	
H22	0.1299	0.3593	0.4623	0.080*	
C23	0.1226 (4)	0.2247 (3)	0.4582 (3)	0.0580 (14)	
C24	0.0921 (6)	0.3799 (4)	0.2929 (5)	0.115 (3)	
H24A	0.0338	0.3952	0.2886	0.173*	
H24B	0.1122	0.3608	0.2414	0.173*	
H24C	0.1238	0.4293	0.3100	0.173*	
C25	0.1364 (5)	0.1919 (4)	0.5424 (4)	0.095 (2)	
H25A	0.0850	0.1676	0.5626	0.142*	
H25B	0.1536	0.2388	0.5764	0.142*	
H25C	0.1793	0.1483	0.5419	0.142*	
C26	0.0658 (4)	0.1895 (4)	0.2552 (3)	0.0679 (16)	
C32	0.2068 (4)	0.1314 (4)	0.2339 (4)	0.0711 (17)	
H32	0.2248	0.1655	0.2767	0.085*	
C31	0.1223 (3)	0.1290 (3)	0.2129 (3)	0.0495 (12)	
C33	0.2646 (4)	0.0820 (5)	0.1902 (4)	0.086 (2)	
H33	0.3213	0.0829	0.2038	0.103*	
C34	0.2379 (5)	0.0334 (5)	0.1288 (4)	0.085 (2)	
H34	0.2763	0.0012	0.0992	0.102*	
C35	0.1495 (5)	0.0305 (4)	0.1079 (3)	0.0738 (18)	
H35	0.1312	−0.0047	0.0659	0.089*	
C36	0.0945 (4)	0.0783 (4)	0.1488 (3)	0.0687 (17)	
C37	0.0019 (5)	0.0762 (5)	0.1242 (4)	0.093 (2)	
H37B	−0.0105	0.1254	0.0910	0.140*	
H37C	−0.0328	0.0776	0.1715	0.140*	
H37A	−0.0092	0.0242	0.0945	0.140*	
N11	0.1224 (3)	−0.0881 (3)	0.4099 (2)	0.0491 (10)	
N12	0.1103 (3)	−0.1256 (3)	0.4824 (2)	0.0521 (10)	
H12A	0.0943	−0.0982	0.5248	0.062*	
N21	0.1115 (2)	0.1715 (2)	0.3970 (2)	0.0425 (9)	
N22	0.0961 (3)	0.2227 (3)	0.3322 (3)	0.0650 (13)	
O1	0.0029 (4)	0.2206 (3)	0.2275 (3)	0.1102 (19)	
Cl1	0.25848 (9)	0.04521 (9)	0.42155 (10)	0.0660 (4)	
Cl2	−0.02466 (9)	0.02924 (9)	0.36848 (9)	0.0614 (4)	
Pd1	0.11619 (2)	0.03965 (2)	0.39821 (2)	0.04292 (14)	

### Atomic displacement parameters (Å^2^)

**Table:**

	*U*^11^	*U*^22^	*U*^33^	*U*^12^	*U*^13^	*U*^23^
C11	0.058 (3)	0.038 (3)	0.068 (3)	0.002 (2)	−0.001 (3)	0.012 (2)
C12	0.064 (3)	0.033 (3)	0.080 (4)	−0.004 (2)	0.005 (3)	−0.006 (3)
C13	0.070 (4)	0.041 (3)	0.054 (3)	0.002 (3)	0.014 (3)	−0.009 (2)
C14	0.110 (6)	0.063 (4)	0.088 (5)	0.006 (4)	0.000 (4)	0.026 (4)
C15	0.156 (7)	0.071 (4)	0.058 (4)	−0.007 (5)	0.025 (4)	−0.009 (3)
C21	0.106 (5)	0.037 (3)	0.074 (4)	0.011 (3)	0.004 (4)	0.001 (3)
C22	0.082 (4)	0.046 (3)	0.072 (4)	0.003 (3)	−0.001 (3)	−0.022 (3)
C23	0.077 (4)	0.043 (3)	0.053 (3)	0.008 (3)	−0.002 (3)	−0.011 (2)
C24	0.182 (9)	0.053 (4)	0.111 (6)	0.023 (5)	0.007 (6)	0.022 (4)
C25	0.162 (8)	0.072 (4)	0.051 (4)	0.014 (5)	−0.011 (4)	−0.012 (3)
C26	0.090 (5)	0.057 (4)	0.056 (3)	0.022 (3)	−0.004 (3)	0.006 (3)
C32	0.078 (4)	0.082 (4)	0.054 (3)	−0.003 (4)	0.002 (3)	0.011 (3)
C31	0.066 (3)	0.043 (3)	0.040 (2)	0.006 (3)	0.004 (2)	0.008 (2)
C33	0.070 (5)	0.109 (6)	0.078 (5)	0.017 (4)	0.018 (4)	0.005 (4)
C34	0.099 (6)	0.089 (5)	0.069 (4)	0.030 (4)	0.028 (4)	0.008 (4)
C35	0.117 (6)	0.056 (4)	0.049 (3)	0.016 (4)	0.013 (3)	0.001 (3)
C36	0.108 (5)	0.053 (3)	0.045 (3)	0.000 (3)	0.010 (3)	0.008 (3)
C37	0.093 (5)	0.096 (5)	0.090 (5)	−0.014 (4)	−0.029 (4)	0.000 (4)
N11	0.065 (3)	0.041 (2)	0.041 (2)	−0.002 (2)	−0.004 (2)	0.0020 (17)
N12	0.065 (3)	0.044 (2)	0.047 (2)	0.006 (2)	0.003 (2)	0.0008 (19)
N21	0.055 (2)	0.034 (2)	0.039 (2)	0.0076 (17)	−0.0045 (19)	−0.0001 (17)
N22	0.107 (4)	0.039 (2)	0.049 (3)	0.010 (2)	−0.006 (2)	−0.003 (2)
O1	0.126 (4)	0.119 (4)	0.085 (3)	0.064 (4)	−0.030 (3)	−0.013 (3)
Cl1	0.0588 (8)	0.0530 (8)	0.0862 (10)	−0.0017 (7)	−0.0193 (7)	0.0089 (7)
Cl2	0.0509 (7)	0.0671 (9)	0.0661 (8)	0.0038 (7)	−0.0010 (6)	−0.0147 (7)
Pd1	0.0545 (2)	0.0340 (2)	0.0403 (2)	0.00246 (17)	−0.00455 (17)	−0.00125 (16)

### Geometric parameters (Å, °)

**Table:**

C11—N12	1.342 (6)	C25—H25C	0.9600
C11—C12	1.358 (8)	C26—O1	1.202 (7)
C11—C14	1.501 (8)	C26—N22	1.459 (7)
C12—C13	1.388 (7)	C26—C31	1.475 (7)
C12—H12	0.9300	C32—C31	1.389 (8)
C13—N11	1.340 (6)	C32—C33	1.398 (8)
C13—C15	1.498 (8)	C32—H32	0.9300
C14—H14A	0.9600	C31—C36	1.394 (8)
C14—H14B	0.9600	C33—C34	1.336 (10)
C14—H14C	0.9600	C33—H33	0.9300
C15—H15A	0.9600	C34—C35	1.449 (10)
C15—H15B	0.9600	C34—H34	0.9300
C15—H15C	0.9600	C35—C36	1.333 (8)
C21—C22	1.328 (9)	C35—H35	0.9300
C21—N22	1.377 (7)	C36—C37	1.529 (9)
C21—C24	1.501 (8)	C37—H37B	0.9600
C22—C23	1.398 (8)	C37—H37C	0.9600
C22—H22	0.9300	C37—H37A	0.9600
C23—N21	1.319 (6)	N11—N12	1.350 (5)
C23—C25	1.503 (8)	Pd1—N11	1.989 (4)
C24—H24A	0.9600	Pd1—N21	2.042 (4)
C24—H24B	0.9600	Pd1—Cl1	2.2981 (15)
C24—H24C	0.9600	Pd1—Cl2	2.3001 (15)
C25—H25A	0.9600	N12—H12A	0.8600
C25—H25B	0.9600	N21—N22	1.359 (5)
			
N12—C11—C12	106.2 (5)	N22—C26—C31	116.0 (5)
N12—C11—C14	121.3 (5)	C31—C32—C33	119.4 (6)
C12—C11—C14	132.5 (5)	C31—C32—H32	120.3
C11—C12—C13	107.1 (5)	C33—C32—H32	120.3
C11—C12—H12	126.5	C32—C31—C36	120.9 (5)
C13—C12—H12	126.5	C32—C31—C26	117.0 (5)
N11—C13—C12	109.3 (5)	C36—C31—C26	121.7 (5)
N11—C13—C15	120.5 (5)	C34—C33—C32	119.6 (7)
C12—C13—C15	130.1 (5)	C34—C33—H33	120.2
C11—C14—H14A	109.5	C32—C33—H33	120.2
C11—C14—H14B	109.5	C33—C34—C35	120.6 (6)
H14A—C14—H14B	109.5	C33—C34—H34	119.7
C11—C14—H14C	109.5	C35—C34—H34	119.7
H14A—C14—H14C	109.5	C36—C35—C34	119.8 (6)
H14B—C14—H14C	109.5	C36—C35—H35	120.1
C13—C15—H15A	109.5	C34—C35—H35	120.1
C13—C15—H15B	109.5	C35—C36—C31	119.6 (7)
H15A—C15—H15B	109.5	C35—C36—C37	118.9 (6)
C13—C15—H15C	109.5	C31—C36—C37	121.5 (6)
H15A—C15—H15C	109.5	C36—C37—H37B	109.5
H15B—C15—H15C	109.5	C36—C37—H37C	109.5
C22—C21—N22	106.5 (5)	H37B—C37—H37C	109.5
C22—C21—C24	131.2 (6)	C36—C37—H37A	109.5
N22—C21—C24	122.2 (6)	H37B—C37—H37A	109.5
C21—C22—C23	107.3 (5)	H37C—C37—H37A	109.5
C21—C22—H22	126.3	C13—N11—N12	105.4 (4)
C23—C22—H22	126.3	C13—N11—Pd1	133.6 (3)
N21—C23—C22	110.0 (5)	N12—N11—Pd1	120.5 (3)
N21—C23—C25	121.6 (5)	C11—N12—N11	112.0 (4)
C22—C23—C25	128.4 (5)	C11—N12—H12A	124.0
C21—C24—H24A	109.5	N11—N12—H12A	124.0
C21—C24—H24B	109.5	C23—N21—N22	105.7 (4)
H24A—C24—H24B	109.5	C23—N21—Pd1	127.7 (3)
C21—C24—H24C	109.5	N22—N21—Pd1	126.6 (3)
H24A—C24—H24C	109.5	N21—N22—C21	110.4 (4)
H24B—C24—H24C	109.5	N21—N22—C26	123.3 (4)
C23—C25—H25A	109.5	C21—N22—C26	125.8 (5)
C23—C25—H25B	109.5	N11—Pd1—N21	174.88 (15)
H25A—C25—H25B	109.5	N11—Pd1—Cl1	88.39 (13)
C23—C25—H25C	109.5	N21—Pd1—Cl1	90.02 (12)
H25A—C25—H25C	109.5	N11—Pd1—Cl2	89.98 (13)
H25B—C25—H25C	109.5	N21—Pd1—Cl2	91.84 (12)
O1—C26—N22	117.9 (5)	Cl1—Pd1—Cl2	176.71 (5)
O1—C26—C31	125.4 (6)		
			
N12—C11—C12—C13	−0.2 (6)	C14—C11—N12—N11	180.0 (5)
C14—C11—C12—C13	−179.9 (6)	C13—N11—N12—C11	−0.1 (6)
C11—C12—C13—N11	0.1 (7)	Pd1—N11—N12—C11	−173.1 (3)
C11—C12—C13—C15	179.5 (7)	C22—C23—N21—N22	−4.3 (6)
N22—C21—C22—C23	−2.1 (7)	C25—C23—N21—N22	176.5 (6)
C24—C21—C22—C23	−179.4 (7)	C22—C23—N21—Pd1	175.7 (4)
C21—C22—C23—N21	4.1 (7)	C25—C23—N21—Pd1	−3.5 (8)
C21—C22—C23—C25	−176.7 (7)	C23—N21—N22—C21	3.0 (6)
C33—C32—C31—C36	−0.2 (8)	Pd1—N21—N22—C21	−177.0 (4)
C33—C32—C31—C26	−173.4 (6)	C23—N21—N22—C26	−168.9 (5)
O1—C26—C31—C32	149.9 (7)	Pd1—N21—N22—C26	11.1 (7)
N22—C26—C31—C32	−21.1 (7)	C22—C21—N22—N21	−0.5 (7)
O1—C26—C31—C36	−23.3 (10)	C24—C21—N22—N21	177.0 (6)
N22—C26—C31—C36	165.7 (5)	C22—C21—N22—C26	171.1 (6)
C31—C32—C33—C34	0.2 (10)	C24—C21—N22—C26	−11.3 (11)
C32—C33—C34—C35	−0.9 (11)	O1—C26—N22—N21	125.6 (7)
C33—C34—C35—C36	1.7 (10)	C31—C26—N22—N21	−62.7 (7)
C34—C35—C36—C31	−1.7 (9)	O1—C26—N22—C21	−45.1 (10)
C34—C35—C36—C37	178.3 (6)	C31—C26—N22—C21	126.6 (6)
C32—C31—C36—C35	1.0 (8)	C13—N11—Pd1—Cl1	−79.2 (5)
C26—C31—C36—C35	173.9 (5)	N12—N11—Pd1—Cl1	91.4 (3)
C32—C31—C36—C37	−179.0 (5)	C13—N11—Pd1—Cl2	97.9 (5)
C26—C31—C36—C37	−6.1 (8)	N12—N11—Pd1—Cl2	−91.5 (3)
C12—C13—N11—N12	0.0 (6)	C23—N21—Pd1—Cl1	−68.9 (4)
C15—C13—N11—N12	−179.5 (6)	N22—N21—Pd1—Cl1	111.1 (4)
C12—C13—N11—Pd1	171.6 (4)	C23—N21—Pd1—Cl2	113.8 (4)
C15—C13—N11—Pd1	−7.9 (9)	N22—N21—Pd1—Cl2	−66.2 (4)
C12—C11—N12—N11	0.2 (6)		

### Hydrogen-bond geometry (Å, °)

**Table:**

*D*—H···*A*	*D*—H	H···*A*	*D*···*A*	*D*—H···*A*
N12—H12A···Cl2^i^	0.86	2.35	3.194 (4)	169

Symmetry codes: (i) −*x*, −*y*, −*z*+1.
